# Identification and bioactive potential of endophytic fungi isolated from selected plants of the Western Himalayas

**DOI:** 10.1186/2193-1801-2-8

**Published:** 2013-01-11

**Authors:** Masroor Qadri, Sarojini Johri, Bhahwal A Shah, Anamika Khajuria, Tabasum Sidiq, Surrinder K Lattoo, Malik Z Abdin, Syed Riyaz-Ul-Hassan

**Affiliations:** 1 Microbial Biotechnology Division, CSIR-Indian Institute of Integrative Medicine, Canal Road, Jammu, 180001 India; 2 Microbial Natural Product Chemistry Division, CSIR-Indian Institute of Integrative Medicine, Canal Road, Jammu, 180001 India; 3 Pharmacology Division, CSIR-Indian Institute of Integrative Medicine, Canal Road, Jammu, 180001 India; 4 Plant Biotechnology Division, CSIR-Indian Institute of Integrative Medicine, Canal Road, Jammu, 180001 India; 5 Department of Biotechnology, Centre for Transgenic Plant Development, Jamia Hamdard, New Delhi 1 10062 India

**Keywords:** Endophytes, Western Himalayas, Fungal diversity, Conifers, Antimicrobial activity, Immuno-modulation, ITS

## Abstract

This study was conducted to characterize and explore the endophytic fungi of selected plants from the Western Himalayas for their bioactive potential. A total of 72 strains of endophytic fungi were isolated and characterized morphologically as well as on the basis of ITS1-5.8S-ITS2 ribosomal gene sequence acquisition and analyses. The fungi represented 27 genera of which two belonged to Basidiomycota, each representing a single isolate, while the rest of the isolates comprised of Ascomycetous fungi. Among the isolated strains, ten isolates could not be assigned to a genus as they displayed a maximum sequence similarity of 95% or less with taxonomically characterized organisms. Among the host plants, the conifers, *Cedrus deodara, Pinus roxburgii* and *Abies pindrow* harbored the most diverse fungi, belonging to 13 different genera, which represented almost half of the total genera isolated. Several extracts prepared from the fermented broth of these fungi demonstrated strong bioactivity against *E. coli* and *S. aureus* with the lowest IC_50_ of 18 μg/ml obtained with the extract of *Trichophaea abundans* inhabiting *Pinus* sp. In comparison, extracts from only three endophytes were significantly inhibitory to *Candida albicans,* an important fungal pathogen. Further, 24 endophytes inhibited three or more phytopathogens by at least 50% in co-culture, among a panel of seven test organisms. Extracts from 17 fungi possessed immuno-modulatory activities with five of them showing significant immune suppression as demonstrated by the *in vitro* lymphocyte proliferation assay. This study is an important step towards tapping the endophytic fungal diversity from the Western Himalayas and assessing their bioactive potential. Further studies on the selected endophytes may lead to the isolation of novel natural products for use in medicine, industry and agriculture.

## Introduction

Microorganisms are important sources of bioactive natural products with enormous potential for the discovery of new molecules for drug discovery, industrial use and agricultural applications (Demain 1999; Keller et al. 2005; Strobel 2006; Porras-Alfaro and Bayman 2011). Natural products remain a consistent source of drug leads with more than 40% of new chemical entities (NCEs) reported from 1981 to 2005 having been derived from microorganisms (Khosla 1997; Clardy and Walsh 2004; Sieber and Marahiel 2005). Further, more than 60% of the anticancer and 70% of the antimicrobial drugs currently in clinical use are natural products or natural product derivatives (McAlpine et al. 2005). This is not surprising in the light of their evolution over millions of years in diverse ecological niches and natural habitats. In comparison to other natural sources like plants, microorganisms are highly diverse but narrowly explored. Studies based on estimation of microbial populations have revealed that only about 1% of bacteria and 5% of fungi have been characterized and the rest remain unexplored for their contribution to the human welfare (Heywood 1995; Staley et al. 1997).

The potential of microorganisms is further limited by the presence of orphan biosynthetic pathways that remain unexpressed under general laboratory conditions (Bok et al. 2006; Hertweck 2009). However, the vast choice of techniques pertaining to the growth and manipulation of microorganisms like media engineering, co-culture, chemical induction, epigenetic modulation and metabolite remodeling, coupled with the fermentation technology for scale up, make them suitable for production of useful natural products, both known and novel (Bok et al*.*^2006^; Bergmann et al. 2007; Knappe et al. 2008; Schroeckh et al. 2009; Riyaz-Ul-Hassan et al*.*^2012^). Thus, it has become imperative to explore microorganisms for NCEs and lead-drug-molecules to run sustainable programs like drug discovery. Consequently, bioprospecting of microorganisms is carried out from every possible source, including extreme environments like ocean beds, geothermal vents, cold desserts etc., in search of novel strains with promising bioactivities (Staley et al. 1997; Selvin et al. 2010; Paul Antony et al. 2012).

During the last 20 years, it has been observed that much of the wealth of microbial biodiversity with novel biochemistry and secondary metabolite production resides in plant tissues (Strobel 2006; Porras-Alfaro and Bayman 2011). Interest in such microorganisms, termed as endophytes, increased immensely with the discovery of an endophytic fungus, from *Taxus brevifolia,* producing the billion dollar anti-cancer drug, taxol (Stierle et al. 1993). Numerous bioactive molecules have been isolated from endophytic fungi since this ground breaking discovery (Strobel 2006; Wang et al. 2011; Zhang et al. 2012). Endophytes are metabolically more active than their free counterparts due to their specific functions in nature and activation of various metabolic pathways to survive in the host tissues (Strobel and Daisy 2003; Strobel 2006; Riyaz-Ul-Hassan et al. 2012). As the previous research on endophytes mainly focused on search for the host-plant metabolites in the endophytic partner (Stierle et al. 1993; Puri et al. 2006; Kusari et al*.*^2009^), the theory of horizontal transfer from the host plant to its microbial symbiont received much impetus (Strobel 2006). However, the sequencing of the *taxadiene synthase* gene from the taxol-producing endophyte revealed that endophytes possess biosynthetic pathways independent of the plant host (Staniek et al. 2009). This indicates that microorganisms have much more biosynthetic proficiency than previously thought. Thus, microorganisms may be screened for a wide range of biological activities and explored for useful chemical entities consistently produced by them.

Establishment of microbial repositories from various ecological niches is an important step towards tapping their potential for human welfare, including drug discovery and sustainable agriculture. The Himalayas and its foothills represent an important biodiversity hot-spot of the world (Hanson et al. 2009). The state of Jammu and Kashmir (J&K) possesses a significant portion of Himalayas (Western Himalayas), with areas of high altitude, cold desserts, glaciers and immense plant diversity including a plethora of medicinal and aromatic plants (Khuroo et al*.*^2007^; Hanson et al. 2009). The microbial resources of the Western Himalayas, particularly the endophytic populations, are mostly unexplored. Thus, an investigation was carried out to isolate and characterize the endophytic fungi of selected plants growing in the Himalayas and its foothills, with the aim to establish a repository, explore their bioactive potential and isolate new leads for drug discovery, industry and agriculture. In this report, we describe the characterization of endophytic fungi obtained from such plants and their bioactive potential with respect to antimicrobial activity and immune modulation.

## Materials and Methods

### Collection of the host plants

Small cuttings (about 10 cm) of the stems or twigs of the plants were collected from specific locations in the Western Himalayas of the Lolab Valley (6000–6500 feet; 34°31'13"N, 74°22'55"E) and Sadhana Top (11,000 feet; 34°24'5"N, 73°57'14"E). Lolab valley is known for its dense Cedrus forests whereas Sadhna top is thickly covered with wild growing *Artemisia* and *Mentha* species in addition to dense coniferous trees and other medicinal plants. Multiple numbers of samples from individual trees (n = 20 for each) of *Cedrus deodara* and *Pinus roxburgii* were collected from both the locations. Samples of *Platanus orientalis* and *Cannabis sativa* were collected from the former location whereas *Abies pindrow,* and *Artemisia annua* were obtained from the latter. Shoot cuttings of *Picrorhiza kurroa* were procured from the Himalayan region of Sonamarg (34°18^′^17^′′^ N, 75°17^′^8^′′^ E). Specimens of other plants, *Withania somnifera, Rauwolfia serpentina* and *Nothapodytes nimmoniana* were collected from the foothills of Shivalik range of Himalayas at Jammu (32°43'54"N, 74°50'53"E). For each host, samples from ten plants were collected except for *Nothapodytes nimmoniana,* wherein samples were collected from a single plant. The plant materials were transported to the lab in sterile polythene bags and stored at 4°C until processed. The authenticated specimen of the collection were deposited in the CSIR-IIIM herbarium, Jammu, India (Table 1).

**Table 1:** **The list of endophytes isolated in this study, their host plants and the summary of BLAST results, showing the coverage of the sequences and sequence similarities with the most closely related organisms** (Altschul et al. 1997)

No.	Plant host; Voucher no.	Endophytes isolated (GenBank Acc. no. of the ITS sequence)	Query coverage	% sequence similarity	Organism with the highest sequence identity, GenBank Acc. no.
1.	***Picrorhiza kurroa;*RRL(H)17772**	PR1 ( JQ769230 )	100	95	*Chaetomium globosum,* GQ365152.1
		PR2 ( JQ769231)	99	99	*Valsa sordida,* HQ420239.1
		PR3 ( JQ769232 )	98	93	*Thielavia subthermophila,* JN390827.1
		PR4 ( JQ769233 )	100	99	*Diaporthe phaseolorum,* EF488429.1
2.	***Cannabis sativa;*RRL(H)18255**	CN1 ( JQ769234 )	100	98	*Alternaria alternata,* JN038476.1
		CN2 ( JQ769235 )	100	99	*Schizophyllum commune,* EU030374.1
		CN3 ( JQ769236 )	99	97	*Alternaria* sp. JN689942.1
		CN4 ( JQ769237 )	100	96	*Alternaria brassicae,* JF439450.1
3.	***Withania somnifera;*RRL(H)22006**	WEF1 ( JQ769238 )	100	99	*Gibberella moniliformis,* GQ168841.1
		WEF2 ( JQ769239 )	100	99	*Cochliobolus lunatus,* GQ328851.1
		WEF3 ( JQ769240 )	100	99	*Fusarium* sp., EU236709.1
		WEF4 ( JQ769241 )	100	99	*Fusarium equiseti,* EU595566.1
		WEF5 ( JQ769242 )	100	98	*Gibberella moniliformis,* JF499680.1
		WEF7 ( JQ769243 )	100	98	*Hypoxylon fragiforme,* JN564001.1
		WEF8 ( JQ769244 )	100	99	*Nigrospora sphaerica,* HQ608030.1
		WEF9 ( JQ769245 )	100	94	*Cercophora caudata,* AY999135.1
		WEF10 ( JQ769246 )	100	95	*Cladosporium cladosporioides,* JN618353.1
4.	***Rauwolfia serpentina;*RRL(H)19053**	RSL1 ( JQ769247)	100	97	*Alternaria brassicae,* JF439438.1
		RSL2 ( JQ769248 )	100	99	*Alternaria* sp. HQ596520.1
		RSL3 ( JQ769249 )	100	98	*Cladosporium cladosporioides,* GQ221853.1
		RSL4 ( JQ769250 )	100	98	*Fusarium proliferatum,* X94171.1
		RSL5 ( JQ769251 )	100	97	*Alternaria brassicae*, JF439444.1
		RSS2 ( JQ769252 )	100	98	*Alternaria alternata,* JN618076.1
		RSS3 ( JQ769253 )	100	97	*Diaporthe helianthi,* AJ312356.1
		RSS4 ( JQ769254 )	100	98	*Fusarium proliferatum,* HQ022511.1
		RSS5 ( JQ769255 )	100	99	*Alternaria* sp. , HQ875381.1
		RSS6 ( JQ769256 )	100	97	*Alternaria alternata,* JN618076.1
		RSS7 ( JQ769257 )	100	97	*Alternaria* sp. , GU934499.1
		RSS9 ( JQ769258 )	100	97	*Lasiodiplodia theobromae,* GQ502453.1
		RSR1 ( JQ769259 )	100	98	*Glomerella acutata,* HM575269.1
5.	***Cedrus deodara;*RRL(H)1832)**	DEF1 ( JQ769260 )	100	98	*Sordaria humana,* EU918705.1
		DEF2 ( JQ769261 )	100	100	*Alternaria alternaria,* JN618076.1
		DEF3 ( JQ769262 )	100	93	*Talaromyces trachyspermus,* AY533702.1
		DEF4 ( JQ769263 )	100	99	*Cochliobolus spicifer,* JN192387.1
		DEF5 ( JQ769264 )	99	98	*Scleroconidioma* s*phagnicola,* FR837912.1
6.	***Abies pindrow;*RRL(H)21551**	FEF2 ( JQ769265 )	99	99	*Daldinia fissa,* AM292038.1
		FEF3 ( JQ769266 )	99	96	*Penicillium oxalicum,* GU078430.1
		FEF4 ( JQ769267 )	99	98	*Polyporus arcularius,* AF516524.1
		FEF5 ( JQ769268 )	98	95	*Apiosordaria otanii,* GQ922524
7.	***Pinus roxburgii;*RRL(H)15011**	K1 ( JQ769269 )	99	99	*Petriella* sp. EU315012.1
		K2 ( JQ769270 )	100	97	*Bipolaris tetramera,* HM195268.1
		K4 ( JQ769271 )	100	94	*Trichophaea abundans* EU715596
		K6 ( JQ769272 )	100	95	*Penicillium expansum,* FJ770072.1
		K7 ( JQ769273 )	99	99	*Ulocladium* sp. JF311922.1
8.	***Nothapodytes nimmoniana;*RRL(H)20702**	NF1 ( JQ769274 )	100	98	*Phomopsis* sp. FJ441623.1
		NF2 ( JQ769275 )	100	93	*Petriella setifera,* AY882356.1
9.	***Platanus orientalis;*RRL(H)19697**	CH2 ( JQ769276 )	100	99	*Fusarium tricinctum,* HM776425.1
		CH3 ( JQ769277 )	100	100	*Fusarium solani,* EU314965.1
		CH5 ( JQ769278 )	100	99	*Fusarium* sp., EU589150.1
		CH6 ( JQ769279 )	100	100	*Gibberella* sp., FJ196601.1
10.	***Artemisia annua; RRL(H)18128)***	Art ( JQ769280 )	100	100	*Fusarium tricinctum,* AB369452.1
		Art1 ( JQ769281 )	100	99	*Fusarium flocciferum,* GQ505465.1
		Art2 ( JQ769282 )	98	98	*Gibberella avenacea,* HM036596.1
		Art3 ( JQ769283 )	100	99	*Sordaria superba,* EU551188.1
		Art4 ( JQ769284 )	100	100	*Fusarium redolens,* JF311916.1
		Art5 ( JQ769285 )	100	99	*Chaetomium* sp., HQ914898.1
		Art6 ( JQ769286 )	100	100	*Alternaria alternata,* GU073112.1
		Art7 ( JQ769287 )	100	99	*Alternaria alternata,* JN618076.1
		Art8 ( JQ769288 )	100	99	*Chaetomium globosum,*GQ365152.1
		Art9 ( JQ769289 )	100	98	*Alternaria* sp., HQ914883.1
		Art10 ( JQ769290 )	100	99	*Alternaria brassicae,* JF439439.1
		Art11 ( JQ769291 )	100	99	*Alternaria* sp., JF694748.1
		Art12 ( JQ769292 )	100	99	*Alternaria* sp., GU934499.1
		Art14 ( JQ769293 )	100	98	*Alternaria brassicae,* JF439444.1
		Art15 ( JQ769294 )	100	99	*Alternaria citri,* DQ339104.1
		Art17 ( JQ769295 )	100	98	*Alternaria brassicae,* JF439444.1
		Art18 ( JQ769296 )	100	100	*Paraphoma* sp., FJ903342.1
		Art19 ( JQ769297 )	98	93	*Alternaria tenuissima,* HQ343444.1
		Art20 ( JQ769298 )	100	98	*Alternaria arborescens,* AY154706.1
		Art23 ( JQ769299 )	88	92	*Alternaria alternata,* JF311960.1
		Art25 ( JQ769300 )	100	99	*Alternaria* sp., JF694748.1
		Art36 ( JQ769301 )	100	96	*Gibberella avenacea,* EU255804.1

### Isolation of endophytes

Endophytic fungi were isolated as described previously by Ezra et al. (2004). Plant materials were thoroughly washed with running tap water, cut under sterile conditions into small pieces (2–3 cm) and surface sterilized with 1% sodium hypochlorite and 90% ethanol, respectively. Before the treatment with alcohol traces of sodium hypochlorite were removed with a rinse in sterile distilled water. The outer tissues were removed after drying the plant tissues under the sterile laminar air flow and passing through the flame. The internal tissues were cut into smaller pieces of 0.5 to 1 cm and plated on different microbiological media such as water agar, potato dextrose agar and rose bengal agar (Difco). The plates were incubated at 25°C for three weeks. Hyphal tips of fungi, emerging out of the plant tissues, were picked and grown on potato dextrose agar in pure culture. After the proper incubation of the plates, seven day old cultures were preserved by placing pieces of hyphal growth in 15% glycerol and storing at −70°C. All the media and chemicals were obtained from Difco (USA). The cultures were also submitted to the IIIM Microbial repository for lyophilization and preservation.

For tentative identification, microscopic slides of each endophyte were prepared by staining with lactophenol-cotton-blue (Vainio et al., 1998) and were examined under light microscope (Olympus, USA).

### Genomic DNA Extraction

Each endophytic fungus was cultured in PD broth at 25°C with constant shaking for 7 days. The fungal mycelia were freeze-dried and the genomic DNA was extracted by the CTAB (Cetyl trimethylammonium bromide) method (Ausubel et al. 1994). Briefly, 500 mg of fungal mycelia were vigorously crushed in liquid nitrogen to make a fine powder. The cells were lysed in 10 ml of extraction buffer (50 mM Tris–HCl pH 8.0, 50 mM EDTA, 0.7 M NaCl, 2% cetrimide, 1% SDS and 50 μl β-mercaptoethanol), mixed thoroughly and incubated at 65°C for 30 min with continuous shaking. The lysate was extracted with an equal volume of chloroform/isoamyl alcohol (24:1) and centrifuged at 10,000 × *g* for 10 min at 4°C. The aqueous phase was transferred to a sterile tube; the genomic DNA was precipitated in a 2× volume of chilled isopropanol and centrifuged at 4°C for 10 min at 10,000 × *g*. The resulting pellet was washed twice with 70% ethanol, air dried and dissolved in 20 μl of sterile Millipore water.

### Phylogenetic analyses by partial ITS1-5.8S-ITS2 ribosomal gene sequencing

Phylogenetic analyses of the endophytes were carried out by the acquisition of the ITS1-5.8S-ITS2 ribosomal gene sequencing. The ITS regions of the fungi were amplified with the universal ITS primers, ITS4 (5′TCCTCCGCTTATTGATATGC3′) and ITS5 (5′GGAAGTAAAAGTCGTAACAA3′), using the polymerase chain reaction (PCR). The PCR conditions used were as follows: initial denaturation at 94°C for 3 min followed by 30 cycles of 94°C for 15 sec., 55°C for 30 sec., 72°C for 45 sec., and a final extension at 72°C for 7 min. The 50 μl reaction mixture contained 1× PCR buffer, 200 μM each dNTP, 1.5 mM MgCl_2_, 10 pmol. of each primer, 1–5 ng of DNA and 2.0 U of *Taq* DNA polymerase. The amplified products (5 μl) were visualized on 1% (w/v) agarose gel to confirm the presence of a single amplified band. The amplicons were purified by Amicon Ultra columns (Millipore, USA) and 20–40 ng were used in a 10 μl sequencing reaction using the Big Dye Terminator sequencing kit (v. 3.1). The forward or the reverse primer (2 pmoles) was used in the cycle sequencing reaction. Twenty five cycles of 96°C for 10 s, 50°C for 5 s and 60°C for 4 min were performed and the extension products were purified by ethanol precipitation, dissolved in 10 μl of HiDi Formamide, incubated at 95°C for 5 min and loaded on ABI Prism 377 Genetic Analyzer (Perkin-Elmer) for sequencing. All the reagents for sequencing were from Applied Biosystems, USA. The amplified products were sequenced and aligned with the sequences in the GenBank by BLASTN program (Altschul et al. 1997) to find out the sequence homology with closely related organisms. Sequences from the closely related organisms were downloaded to construct the phylogenetic trees. Endophytes showing complete sequence homology to each other and belonging to the same plant sample were treated as a single isolate. The ITS1-5.8S-ITS2 sequence for each strain was submitted to the GenBank. Accession numbers of the respective strains are provided in Table 1.

### Determination of antimicrobial activities of endophytic extracts

All endophytic cultures were cultivated at 25°C for 15 days in 200 ml of PD broth with constant shaking in an incubator shaker (New Brunswick, USA). The fermentation broth of each endophyte was homogenized with 10% methanol, extracted thrice with methylene chloride, concentrated and dissolved in DMSO (dimethyl sulphoxide) at a concentration of 10 μg/μl. The extracts prepared from the endophytes were evaluated for antimicrobial activity against *Escherichia coli* ATCC 25922 (Gram negative), *Staphylococcus aureus* ATCC 29978 (Gram positive) and *Candida albicans* MTCC 4748 (fungal pathogen)*.* Stock solutions of each culture were prepared in Normal Saline Solution (0.85% NaCl (w/v)) at a concentration of 10^8^ cells/ml. 200 μl Mueller-Hinton Broth (for *E. coli* and *S. aureus*) and PD broth (for *C. albicans*) were added to each well in the first column and 100 μl of corresponding media were added to the rest of the wells. Extracts of different endophytes were added to the first row at a concentration of 100 μg/ml and serially diluted to make concentrations of 50, 25, 12.5 and 6.25 μg/ml. Each well was inoculated with 10^4^ cells of the relevant test organism and incubated at 37°C for 24 h. Each plate had a set of controls: a column with broad-spectrum antibiotics (ciprofloxacin and nystatin) as positive controls, a column without the test extract, and one without the relevant test organism. Absorbance was measured spectrophotometrically at 620 nm and IC_50_ of the extracts was calculated from the average percent inhibition of three replicates of each concentration (Ellof 1998)**.**

### Determination of antimycotic activity of the endophytes

The endophytes were tested against several plant pathogenic fungi (listed in Table 2) to assess the antifungal potential of their extrolites. Briefly, small plugs (ca. 3 mm diameter) of each test fungus were placed a centimeter away from the edge of a 7 days old endophytic culture. The plates were wrapped with Parafilm and incubated at 25°C for 24–48 h. Inhibition of the test pathogens were reported as % inhibition as compared to their relevant controls (Ezra et al*.*^2004^).

**Table 2:** **Antimycotic activity, represented as the% growth inhibition, of the endophytes against several plant pathogens**

S. No.	Endophyte	% Growth inhibition						
		***Fusarium oxysporum*** MTCC 1755	***Aspergillus flavus*** MTCC 1783	***Geotrichum candidum*** MTCC 3993	***Verticillium dahlia*** MTCC 1351	***Fusarium solani*** MTCC 350	***Ceratocystis fimbriata*** MTCC 2281	***Rhizoctonia solani*** MTCC 4633
1.	PR4	41.3 ±2. 8	39.0 ±1.7	54.0 ±3.4	0.0 ±0.0	60.0 ±0.0	57.3 ±2.3	100.0 ±0
2.	DEF3	77 ±4.0	65.6 ±1.1	51.6 ±2.8	52 ±3.4	69.4 ±0.8	70.9 ±3.5	0.0 ±0.0
3.	DEF4	72.6 ±2.3	12.6 ±2.3	40 ±5.1	48.3 ±2.8	71.0 ±2.0	71.3 ±3.2	0.0 ±0.0
4.	RSL1	41.7 ±0.0	25.2 ±0.5	50.2 ±0.9	0.0 ±0.0	34.6 ±0.3	71.5 ±0.0	70.6 ±1.2
5.	RSR1	31.0 ±5.1	43.6 ±6.0	16.3 ±1.1	0.0 ±0.0	69 ±1.7	100 ±0.0	55.3 ±2.3
6.	WEF1	74.8 ±1.9	80 ±0.0	58.8 ±1.0	0.0 ±0.0	63.3 ±1.5	89.9 ±0.8	0.0 ±0.0
7.	WEF2	73.0 ±0.0	65.3 ±2.8	40.0 ±0.0	0.0 ±0.0	73.0 ±0.0	92.6 ±0.5	68.3 ±2.8
8.	WEF4	73.0 ±0.0	51.1 ±3.4	36.6 ±5.7	0.0 ±0.0	65.4 ±1.5	64.3 ±2.3	50.0 ±0.0
9.	WEF9	67.0 ±0.0	23.6 ±3.3	36.6 ±2.7	0.0 ±0.0	56.6 ±3.5	56 ±1.7	31.6 ±2.7
10	WEF10	60.0 ±0.0	31.0 ±5.1	20.0 ±0.0	0.0 ±0.0	54.6 ±2.0	51.6 ±2.8	37.0 ±2.6
11	Art 18	73.0 ±0.0	51.6 ±4.0	40.0 ±0.0	0.0 ±0.0	63.0 ±3.1	56.0 ±1.7	46.6 ±5.7
12	K2	50.0 ±0.0	31.0 ±1.7	19.6 ±0.5	0.0 ±0.0	59.0 ±0.9	70.0 ±0.0	65.0 ±0.5
13	K4	41.7 ±0.2	32.5 ±1.2	57.0 ±0.9	0.0 ±0.0	0.0 ±0.0	88.6 ±0.0	50.0 ±1.3
14	K5	41.7 ±0.2	32.5 ±1.2	57.0 ±0.9	0.0 ±0.0	0.0 ±0.0	88.6 ±0.0	60.0 ±1.0
15	K6	55.6 ±1.1	52.8 ±2.2	60.0 ±1.8	0.0 ±0.0	0.0 ±0.0	88.6 ± 0.0	60.0 ±0.7
16	CN1	25.0 ±0.9	23.1 ±0.6	65.1 ±1.1	0.0 ±0.0	52.0 ±1.4	88.6 ± 0.0	50.0 ±0.4
17	CN2	75.0 ±2.1	31.0 ±1.4	12.0 ±0.2	0.0 ±0.0	60.0 ±1.0	87.0 ±0.7	90.0 ±3.2
18	Art	50.0 ±1.7	53.8 ±0.6	54.3 ±0.4	0.0 ±0.0	60.0 ±0.7	85.7 ±0.2	50.2 ±1.4
19	Art1	50.0 ±1.2	30.8 ±1.5	50.0 ±0.2	0.0 ±0.0	46.2 ±1.3	71.5 ±0.5	53.3 ±1.2
20	Art2	58.4 ±1.7	30.8 ±1.3	50.0 ±0.7	0.0 ±0.0	57.3 ±1.2	72.2 ±2.8	60.0 ±0.9
21	Art4	42.0 ±1.3	28.6 ±0.3	50.0 ±0.6	0.0 ±0.0	55.1 ±0.5	85.7 ±1.6	59.4 ±0.5
22	Art6	50.0 ±0.0	38.5 ±1.3	65.0 ±1.1	0.0 ±0.0	30.0 ±0.3	88.6 ±0.0	65.2 ±0.4
23	Art7	52.0 ±0.3	38.5 ±1.3	64.2 ±1.3	0.0 ±0.0	33.1 ±0.5	88.6 ±0.0	68.3 ±0.7
24	Art9	50.0 ±2.2	25.0 ±1.9	65.3 ±2.3	0.0 ±0.0	50.0 ±0.3	88.6 ±0.0	71.0 ±0.3

Data of endophytes possessing significant activity (50% inhibition or more) against three or more pathogens is presented in this table.

### Determination of Immonomodulatory activity by in vitro lymphocyte proliferation assay

Cell proliferation was measured by MTT assay as described earlier (1983). Splenocytes (2 × 10^6^ cells) were seeded into a 96-well flat-bottom microtiter plate in 100 μl complete medium. Variable doses of test extracts (1 μg and 10 μg), along with Con A (2.5 μg/well) to stimulate T-cell mitogenesis or LPS (2.5 μg/well) to stimulate B-cell mitogenesis, were added making a final volume of 200 μl. The plates were incubated at 37°C with 95% humidity and 5% CO_2_ in a CO_2_ incubator for 72 hrs. 50 μl of MTT solution (5 mg/ml) was added to each well and the plates were incubated for 4 h. The untransformed MTT was removed after centrifugation at 1400 *× g* for 5 min. 200 μl of DMSO: 1 N HCL (24:1) was added to each well, and the absorbance was determined in an ELISA reader at 570 nm after 15 min. All experiments were performed in triplicate and the results were expressed as mean ± S*.*D*.* Student’s *t*-test was used to analyze statistical significance of the differences between the control and the treated values.

## Results

### Phylogenetic affinities of the endophytic fungi

**Figure 1:**
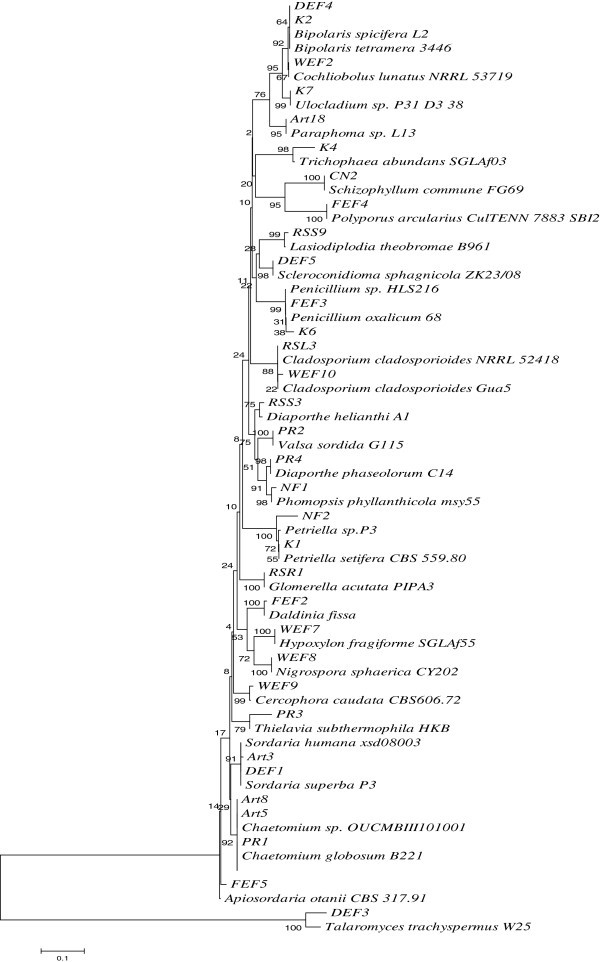
**Phylogenetic relationship between different fungal endophytes, isolated in this study, and their close relatives.** The evolutionary history was inferred using the Neighbor-Joining method (Saitou and Nei 1987). The optimal tree with the sum of branch length = 2.89592492 is shown. The percentage of replicate trees in which the associated taxa clustered together in the bootstrap test (500 replicates) are shown next to the branches (Felsenstein 1985). The tree is drawn to scale, with branch lengths in the same units as those of the evolutionary distances used to infer the phylogenetic tree. The evolutionary distances were computed using the Maximum Composite Likelihood method (Tamura et al. 2004) and are in the units of the number of base substitutions per site. All positions containing gaps and missing data were eliminated from the dataset (Complete deletion option). There were a total of 192 positions in the final dataset. Phylogenetic analyses were conducted according to Tamura et al*.* (2007).

**Figure 2:**
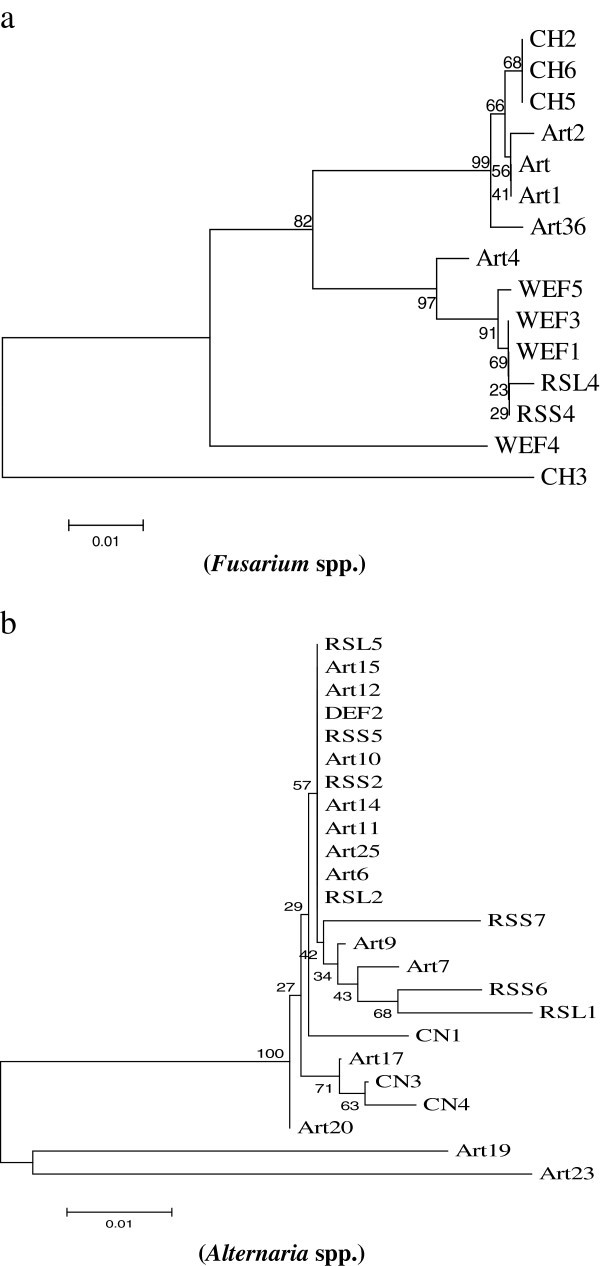
**Phylogenetic relationship between different isolates of*Fusarium*spp.(a)** and *Alternaria* spp. **(b)** inferred using the Neighbor-Joining method. The optimal trees with the sum of branch lengths = 0.22208797 and 0.18904166, respectively, are shown (Tamura et al. 2007).

The acquisition of ITS1-5.8S-ITS2 sequence data and their analyses showed diverse taxonomic affinities among the isolated endophytes**.** In a total of 72 endophytic strains identified in this study, only two Basodiomycete isolates (CN2 and FEF4) were obtained in comparison to 70 isolates of Ascomycetes (Table 1). The sequence of CN2, isolated from *Cannabis sativa,* showed 99% similarity to *Schizophyllum commune* and that of FEF4, isolated from *Abies pindrow* displayed a homology of 98% (99% sequence coverage) with *Polyporus arcularius.* On microscopic analyses, both these isolates were found to produce sterile mycelia on PDA. These two genera make a separate clade in the phylogenetic analysis (Figure 1). The genera of ascomycetous fungi belonged to Sordariomycetes (n = 14), Dothidiomycetes (n = 7), Eurotiomycetes (n = 2), Pezizomycetes (n = 1) and Mitosporic ascomycota (n = 1), in the decreasing order of incidence. These isolates comprised of 25 genera with highest abundance of *Alternaria* spp. (24 isolates) and *Fusarium* spp. (15 isolates, including *Gibberella*), respectively. These two genera constituted more than half (54.2%) of the strains isolated. The highest number of *Alternaria* strains (13/22) was isolated from *Artemisia annua* and *Rauwolfia serpentina* (7/13). Samples of *Platanus orientalis* were found to harbor only *Fusarium* spp. representing 4 different strains. Strangely, strains belonging to other genera could not be obtained from any of the samples of this plant. *Artemesia annua* also possessed several strains of *Fusarium* as endophytes (5/22) whereas almost half of the endophytes of *Withania somnifera* (4/9) were also *Fusarium* spp. Interestingly, the endophytes obtained from the conifers, *Cedrus* sp.*, Pinus* sp. and *Abies* sp. presented significant taxonomic variations. These hosts harbored 13 different genera collectively, among a total of 27 obtained from all the ten plant species. *Picrorhiza kurroa* also harbored four endophytic strains showing homology with four different fungal genera. A total number of ten (13.9%) isolates showed a sequence similarity of 95% or less with known organisms in the GenBank. Further, these isolates produced sterile mycelia on agar plates and thus could not be identified to the species or genus levels. Figure 2 shows the phylogenetic positions of *Fusarium* (a) and *Alternaria* (b) isolates. These two genera comprised of 10 and 6 species, with 15 and 24 numbers of isolates, respectively. The isolates showed significant variations among their ITS sequences. Isolates of *Fusarium* make 4 different clusters, two of which form multiple clades. WEF4 (*Fusarium equiseti*) and CH3 (*Fusarium solani*) each make a separate outgroup. Likewise, endophytic isolates of *Alternaria* make 7 different groups. A group of 12 isolates cluster together showing comparatively low taxonomic variations, whereas two other groups with five and three taxa show considerable sequence divergence. Again, CN1 (*Alternaria alternata*), Art19 (93% sequence homology and 98% sequence coverage with *Alternaria tenuissima*), Art20 (*Alternaria arborescens*) and Art 23 (92% sequence homology and 88% sequence coverage with *Alternaria alternata*) make separate outgroups.

### Antimicrobial activity of the extracts

Extracts from 29 endophytes showed an IC_50_ of less than 100 μg/ml against one or more of the test pathogens (Table 3). A total of seven and eight extracts inhibited *E. coli* and *S. aureus* with an IC_50_ of 50 μg/ml or less, respectively. Extracts from three endophytes, K4 (94% sequence similarity with *Trichophaea abundans* from *Pinus* sp.), PR4 (*Diaporthe phaseolorum* from *Picrorhiza* sp.) and Art4 (*Fusarium redolens* from *Artemisia* sp.) inhibited *S. aureus* strongly with an IC_50_ of 18, 31 and 25, respectively. Two extracts, Art8 (*Chaetomium globosum* from *Artemisia* sp.) and NF1 (*Phomopsis* sp. from *Nothapodytes* sp.) showed an IC_50_ of around 50 μg/ml against both the bacterial pathogens. In comparison, extracts from only three endophytes, Art (*Fusarium tricinctum*), Art2 (*Gibberella avenacea*) and Art9 (*Alternaria* sp.), all hosted by *Artemisia annua,* were significantly active against the fungal pathogen, *Candida albicans* with an IC_50_ of 50, 15 and 50 μg/ml, respectively.

**Table 3:** **Antimicrobial activity of DCM (Dichloromethane) extracts of the endophytes against the bacterial strains,*E. coli*ATCC 25922 (Gram negative),*S. aureus*ATCC 29978 (Gram positive) and*C. albicans*MTCC 4748 (a fungal pathogen)**

S.No.	Code	IC_50_(μg/ml)		
		***Escherichia coli*** ATCC 25922	***Staphylococcus aureus*** ATCC 29978	***Candida albicans*** MTCC4748
1.	Art	-	-	50
2.	Art1	-	88	-
3.	Art 2	-	-	15
4.	Art 4	-	30	-
5.	Art6	56	92	-
6.	Art7	50	-	-
7.	Art 8	50	50	-
8.	Art9	-	-	50
9.	DEF3	-	80	-
10.	K4	95	18	-
11.	K6	-	92	-
12.	K7	-	98	-
13.	FEF2	52	70	-
14.	NF1	50	53	-
15.	NF2	98	-	-
16.	PR1	-	92	-
17.	PR2	90	-	-
18.	PR3	90	88	-
19.	PR4	88	25	-
20.	CN1	78	-	-
21.	CN2	48	-	-
22.	CN3	88	-	-
23.	RSL1	-	92	-
24.	RSL2	-	75	-
25.	RSL3	80	80	-
26.	RSL4	85	50	-
27.	RSL5	50	-	-
28.	RSR1	80	43	-
	Control	0.10 (Ciprofloxacin)	0.09 (Ciprofloxacin)	8.0 (Nystatin)

Among all the endophytes included in this study, significant numbers (29 cultures) displayed an IC_50_ less than 100 μg/ml against one or more of these test organisms.

### Bioactivity against plant pathogens

All the endophytes were evaluated for antimycotic activity against a panel of seven important plant pathogens. 24 endophytes inhibited three or more pathogens by 50% or more (Table 2). 6 isolates (DEF3, WEF1, WEF2, Art, Art2 and Art9) were found highly active inhibiting five of the seven plant pathogens. These organisms belonged to (or were related to) the genera, *Talaromyces* sp.*, Giberella* sp.*, Cochliobolus* sp.*, Fusarium* sp. and *Alternaria* sp*.* Among these were also the isolates, Art, Art2 and Art 9, the only strains active against *C. albicans.*

### *In vitro* lymphocyte proliferation by the extracts

Significant stimulation or inhibition of lymphocytes *in vitro,* with dose response relationship, was demonstrated by extracts from 17 endophytes (Table 4). A total of 5 extracts, K1, K7 (*Petriella* sp. and *Ulocladium* sp., respectively, from *Pinus roxbergii*), DEF4 (*Cochliobolus spicifer* from *Cedrus deodara*) and Art3 and Art4 (*Sordaria superba* and *Fusarium redolens*, respectively, from *Artemisia* sp.) were found to have immunosuppressive properties. Highest inhibition of T-cell proliferation (44%) was obtained at 1 μg/ml of the Art3 extract whereas 10 μg/ml of the same extract resulted in comparatively lower inhibition (37%). However, the extract showed higher inhibition in B-cell proliferation at 10 μg/ml (52%) in comparison to that at 1 μg/ml (38%). The K1 extract potentially inhibited B-cell proliferation (54%) at 1 μg/ml whereas increasing the concentration to 10 μg/ml decreases the activity (22% inhibition). In contrast to immune suppression, more extracts (n = 11) possessed properties that increased proliferation of lymphocytes in a range of 35-58% when compared with the control.

**Table 4:** **A summary of*in vitro*immunomodulatory screening of the endophytic fungal extracts by the lymphocyte proliferation assay**

S.No.	Test Extract	Dose μg/ml	Immunomodulatory activity (% lymphocyte proliferation) Inhibition (↓) /stimulation (↑)	
	Cont		T -Cell	B-Cell
		-	-	-
1.	K1	1	34↓*	54↓**
		10	31↓	22↓
2.	K2	1	15	46↑
		10	12	49↑
3.	K4	1	31↑	40↑
		10	42↑	57↑
4.	K7	1	40↓**	37↓*
		10	25↓	46↓**
5.	DEF3	1	32↑	65↑**
		10	37↑	48↑
6.	DEF4	1	34↓	51↓
		10	34↓	44↓
7.	Art	1	28↑	47↑
		10	33↑	22↑
8.	Art3	1	44↓*	38↓*
		10	37↓*	52↓**
9.	Art4	1	25↓	51↓
		10	40↓**	45↓**
10.	FEF2	1	56↑	40↑*
		10	29↑	47↑**
11.	FEF4	1	57↑**	11
		10	62↑**	18
12.	NF2	1	14↑	32↑*
		10	28↑	45↑
13.	PR1	1	51↑**	19↑
		10	34↑	14↑
14.	PR2	1	15↑	48↑**
		10	28↑	34↑
15.	PR3	1	48↑	43↑
		10	28↑	54↑**
16.	PR4	1	25↑	15↑
		10	16↑	44↑*
Betamethasone		0.005	46↓	34↓
Levamisole		0.05	29↑	43↑

16 extracts demonstrated significant immune-modulation. Levamisole (as stimulator) and Betamethasone (as an immunesuppressor) were used as positive controls. Data are presented as % inhibition or stimulation of sets of independent experiments. * P<0.05 and ** P<0.01 represent significant difference compared with cells treated with Levamisole and Betamethasone.

## Discussion

In this study, endophytic fungi from ten plants growing in the specified Himalayan regions were studied for their phylogenetic affinities and bioactive potential. The plants were selected on the basis of longevity in case of conifers and *Platanus* sp., and medicinal properties for rest of the plants. The endophytes displayed diverse taxonomic positions and bioactive potential. Endophytes from conifers (*Cedrus deodara, Pinus roxbergii* and *Abies pindrow*) possessed a broad range of fungal endophytes, harboring about half of the total genera. These hosts produce bioactive essential oils (Sharma et al. 2008; Kim et al. 2012) that may generate considerable selection pressure for the microbes to colonize inside the plant tissues. They can also survive for several hundred years (Singh and Yadav 2007; Yadav 2009); thus their microbial symbionts may undergo considerable evolutionary changes as the host grows and produces a variety of secondary metabolites during various stages of its life cycle. Diverse endophytes have been isolated by other workers from *Abies* sp. and *Pinus* sp. (Yuan et al. 2011; Ganley and Newcombe, 2006). Such host plants, therefore, may represent important ecological niches for novel microbial strains with useful bioactivities.

*Alternaria* and *Fusarium* species were the dominant taxa obtained from *Withania somnifera, Artemisia annua, Platanus orientalis* and *Rauwolfia serpentina*, as compared to the other host plant. This indicates that endophytic fungi may preferentially colonize plants hosts. However, other factors like soil conditions, climate and the dynamics of soil microflora may also influence the colonization of endophytes in the plant tissues. Although, both of these organisms are fairly explored, they still have the potential to contribute to natural product research as novel bioactive molecules continue to pour in from several of their isolates (Qin et al*.*^2009^; Xu et al. 2010; Mohana et al. 2012). However, it is important to use de-replication techniques to select diverse strains for natural product isolation. One of the most useful information is the inference of phylogenetic relationships based on DNA sequences of ITS regions (Bellemain et al. 2010). Such information is helpful for selection of unique strains based on percent identity and divergence between different strains.

The isolates demonstrating 95% or lesser sequence homology with the known organisms may be preferentially selected for natural product isolation. In this study, 13.9% strains represented unknown genera. In a similar study on the endophytes of *Abies* sp., 27.4% isolates belonged to unknown species (Yuan et al. 2011). Hence, plant tissues inhabit diverse fungi awaiting characterization and exploration. Taxonomic novelty may more likely lead to novel chemistry thus facilitating the isolation of new molecules or NCEs.

Extracts from only three fungi, *Fusarium tricinctum, Gibberella avenacea* and *Alternaria* sp., all endophytes of *Artemisia,* displayed considerable antifungal activities against *C. albicans.* These strains also inhibited several phytopathogens significantly. Some strains of *Fusarium tricinctum* are known to produce different enniatins which have strong biological activities including antifungal properties (Meca et al. 2010). *Gibberella avenacea* is the teleomorph of *Fusarium avenaceum*. Strains of the latter have also been found to produce metabolites like enniatins and moniliformin (Booth and Spooner 1984; Uhlig et al. 2007). It remains to be investigated whether these organisms produce any novel secondary metabolites. Thus, these organisms are being explored for their secondary metabolites. In contrast, several other endophytes were active against only plant pathogenic fungi in co-culture. These organisms need to be studied in detail and may be exploited for disease management for important agricultural crops.

Extracts of five fungi showed significant immune suppression in the *in vitro* lymphocyte proliferation experiments. It is interesting to know that these fungi, possessing immune suppressing activities, belong to five different genera, thus increasing the chances of obtaining different chemical entities when explored for natural product isolation. Despite the availability of effective immunosuppressive drugs in the market, their inherent toxic effects prompts search for alternative immunosuppressants (Cooper and Wiseman, 2010). Fungi are the preferable sources of such molecules owing to the fungal origin of cyclosporins and the fact that they are highly unexplored (Twentyman 1992; Staley et al. 1997). Endophytes from some of the plants explored in this study have been studied earlier with the aim to isolate the host metabolites from the microbial partner (Puri et al. 2007; Rehman et al. 2009). However, our aim is not to limit the endophytes for the exploration of their host metabolites but look into wide applications of their strains including characterization of their novel secondary metabolites.

Endophytes, selected on the basis of bioactivity and phylogenetic novelty, are under investigation for the production of useful bioactive compounds (including VOCs and anticancer compounds) and new molecules with substantial success (data not included). The study indicates that several of these plants support wide spectrum of endophytes with significant bioactive potential. Thus, concerted efforts should be carried out for bioprospection in the Western Himalayas to tap and conserve the microbial resources of this important biodiversity hotspot and utilize their potential for human welfare. In addition, the endophytic populations of these plants may be studied in detail with an ecological perspective which may help to understand community structure of their endophytes and warrant isolation of diverse endophytic fungi with useful bioactivities.
